# Microwave treatment of the cornea leads to localised disruption of the extracellular matrix

**DOI:** 10.1038/s41598-018-32110-0

**Published:** 2018-09-13

**Authors:** Siân R. Morgan, Osamu Hieda, Yoshinori Nakai, Craig Boote, Sally Hayes, Shigeru Kinoshita, Keith M. Meek, Andrew J. Quantock

**Affiliations:** 1 Structural Biophysics Group, School of Optometry and Vision Sciences, College of Biomedical and Life Sciences, Cardiff University, Maindy Road, Cardiff, CF24 4HQ Wales United Kingdom; 2 Cardiff Institute of Tissue Engineering and Repair, School of Pharmacy and Pharmaceutical Sciences, Cardiff University, Redwood Building, King Edward VII Avenue, Cardiff, CF10 3NB United Kingdom; 3 Baptist Eye Hospital, 12 Kamiikeda-cho, Kitashirakawa, Sakyo-ku, Kyoto, 606-8287 Japan; 4 Department of Ophthalmology, Kyoto Prefectural University of Medicine, Hirokoji Kawaramachi, Kamigyo-ku, Kyoto, 602-0841 Japan; 5 Department of Frontier Medical Science and Technology for Ophthalmology, Kyoto Prefectural University of Medicine, Hirokoji Kawaramachi, Kamigyo-ku, Kyoto, 602-0841 Japan

## Abstract

Microwave keratoplasty is a thermo-refractive surgical procedure that can correct myopia (short-sightedness) and pathologic corneal steepening by using microwave energy to cause localised shrinkage around an annulus of the cornea leading to its flattening and vision correction. The effects on the corneal extracellular matrix, however, have not yet been evaluated, thus the current study to assess post-procedure ultrastructural changes in an *in-vivo* rabbit model. To achieve this a series of small-angle x-ray scattering (SAXS) experiments were carried out across whole transects of treated and untreated rabbit corneas at 0.25 mm intervals, which indicated no significant change in collagen intra-fibrillar parameters (i.e. collagen fibril diameter or axial D-period), whereas inter-fibrillar measures (i.e. fibril spacing and the degree of spatial order) were markedly altered in microwave-treated regions of the cornea. These structural matrix alterations in microwave-treated corneas have predicted implications for corneal biomechanical strength and tissue transparency, and, we contend, potentially render microwave-treated corneas resistant to surgical stabilization using corneal cross-linking procedures currently employed to combat refractive error caused by corneal steepening.

## Introduction

The correction of refractive visual dysfunction owing to aberrant eyeball geometry and/or corneal shape is most often carried out by removing predetermined amounts of the corneal stroma to reshape the cornea. Nowadays this is typically achieved via laser photoablation or incisional surgery. Another, less widely used approach of refractive correction, is thermokeratoplasty (TKP) which uses thermal energy to change the shape of the cornea. The basis of this treatment dates back to 1889 when Lans found that the application of radial burns to rabbit cornea resulted in alterations to the cornea’s refractive power^1^. Over the years technological advances have led to new ways of delivering thermal energy to the cornea, and this has generated a sporadic renewed interest in TKP, as comprehensively reviewed by Koch^2^.

TKP, which uses a treatment temperature of 65 °C, is underpinned by the fact that collagen fibrils remain intact, but shorten, when heated to temperatures above 55 °C. This is attributed to the thermally induced modification of the collagen molecules and intermolecular bonds within the collagen fibrils^3–8^. However, careful control of temperature is essential, as overheating can be counterproductive and cause the bonds between the collagen molecules to be broken, and the thermally contracted collagen fibrils to relax^8,9^. TKP involves subjecting corneal collagen (outside of the central 6 mm optical zone^1^) to transiently elevated temperatures. As collagen fibrils are the main structural element of the cornea, their shrinkage has the knock-on effect of altering the cornea’s overall shape, including that of the non-heated optical zone. Over the years corneal heating for TKP has been performed with contact and non-contact lasers^2,10–12^, hot sources^13^, and radiofrequency^14–19^. While the approach has been used, often successfully, to alter the eye’s refractive status to manage presbyopia and correct low-to-moderate hyperopia, it is commonly associated with problems such as regression, lack of predictability, surgically induced astigmatism and higher-order optical aberrations^6^.

As mentioned above, many TKP approaches are designed to treat the corneal mid-periphery, resulting in an annular contraction and corresponding steepening of the central cornea^2^. An alternative microwave-based TKP concept has also been proposed as a potential treatment for mild to moderate myopia, corneal ectasia and keratoconus in a procedure known as microwave keratoplasty^20^. Microwave keratoplasty is carried out using a microwave applicator that is positioned on the eye so that contact is made with the epithelium. Owing to the design of the applicator an annulus of microwave energy is delivered to the paracentral cornea via a dielectrically shielded microwave emitter. The single energy pulse elevates the temperature of the selected region of the stroma to the axial shrinkage temperature of corneal collagen through a means of energy transfer referred to as capacitive coupling. The electric field lines are concentrated in the anterior stroma and the induced electric field causes a rotational oscillation of dipolar water molecules at the microwave frequency, which generates frictional heating^21^. The epithelium and Bowman’s membrane are protected from thermal damage by the system’s evaporative cooling technique, which cools the corneal surface. Stromal collagen undergoes shrinkage in the heated zone, and a toroidal-like lesion is generated in the uppermost 150 µm of the corneal stroma. This lesion induces an annular expansion, rather than contraction, of the treated paracentral zone of the cornea, leading to central corneal flattening and, hence, a refractive correction. The amount of refractive change is a function of the lesion’s diameter and cross-section, and the procedure can yield corrections between roughly 1.00 D and 5.00 D depending on the intensity and configuration of the microwave energy^14^. Despite there being a number of published studies about the effect of various heating modalities on corneal collagen^2,13,16,18,22–24^, knowledge of the effect of microwave treatment is lacking. The current study was thus undertaken to evaluate ultrastructural changes in the stromal matrix following experimental microwave keratoplasty in an *in vivo* rabbit model using small-angle x-ray scattering (SAXS) to provide structural information about the size and spatial arrangement of collagen fibrils within the cornea^25,26^.

## Methods

### Surgical Procedures

All treatments described in this study were performed in accordance with the ARVO Statement for the Use of Animals in Ophthalmic and Vision Research. The experimental protocols were approved by the Kyoto Prefectural University of Medicine Ethics Committee and assigned the decision notification number RBMR-C-622-2. Microwave keratoplasty was performed on one eye of four anaesthetised adult New Zealand White rabbits using a Vedera KXS machine (Avedro Inc. USA). The treatment, designed to correct myopia of approximately −6 D, involved delivery of microwave energy with a frequency of 915 MHz to the cornea in an annulus pattern which measured 3.8 mm (inner diameter) to 4.3 mm (outer diameter). All treated eyes received antibiotic eye drops following the procedure. The four untreated contralateral eyes acted as controls. Corneal topography was performed on the treated and untreated eyes using a topographic modelling system (Tomey, Japan) before treatment, two weeks post-treatment and five weeks post-treatment, and average K values (i.e. measurements of the corneal radius of curvature) were recorded. Five weeks after surgery the animals were euthanized by an intravascular injection of pentobarbital sodium (100 mg/kg), and the corneas with a narrow scleral rim were harvested and fixed in 4% paraformaldehyde to preserve collagen ultrastructure. The microwave annulus in the corneas was clearly visible five weeks after surgery and post-removal fixation in all four treated specimens. The corneal samples were then transported to Cardiff University under refrigerated conditions and stored at 4 °C before x-ray experiments were conducted.

### Small-angle x-ray scattering (SAXS)

**Figure 1:**
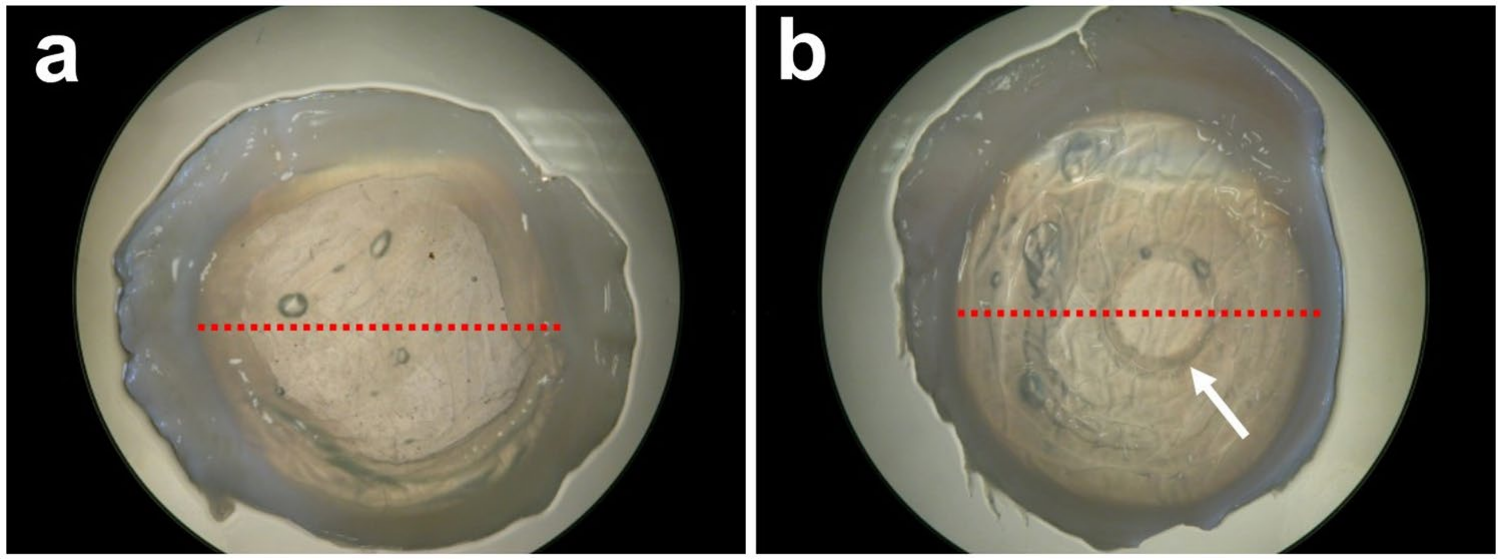
X-ray beam linear scans. Each red dot corresponds to a sampling location on control (**a**) and microwave treated **(b**) rabbit corneas. The scans traversed the discernible microwave region (arrow) at two points.

Microwave treated corneas and controls were transported to the Diamond Light Source national synchrotron facility (Oxfordshire, UK) for SAXS data collection on Beamline I22. Each cornea was wrapped in polyvinylidene chloride catering film to minimize tissue dehydration and positioned in an airtight Perspex (Databank, UK) specimen chamber with a Mylar (Dupont-Teijin, UK) window. SAXS patterns were recorded at 0.25 mm intervals across the centre of each specimen from limbus to limbus, thereby encompassing the discernible microwave annulus of treated specimens and the respective region of controls as indicated in Fig. 1. Given that the limbus-limbus distance of each sample was 12–13 mm, between 48 and 52 SAXS patterns were collected per corneal sample. The x-ray scatter patterns were generated using an x-ray beam with a wavelength 0.1 nm and a cross-sectional diameter 0.2 mm, and a 6 m long x-ray camera. The collagen fibril D-period of hydrated rat tail tendon was used to calibrate the system, and SAXS patterns were analysed according to previously described procedures^27^ to quantify centre-to-centre collagen fibril Bragg spacing, collagen fibril diameter, collagen fibril disorder index and the axial fibrillar D-period, all as an average through the full thickness of the cornea at each sampling location. Data were plotted against relative distance (mm) from the corneal centre.

**Figure 2:**
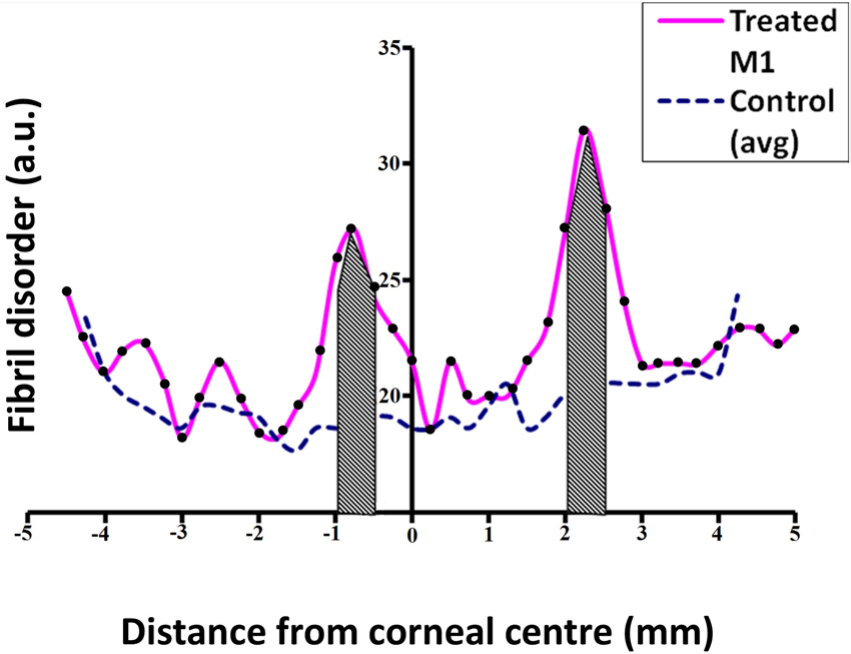
An example parameter profile for collagen fibril disorder. The 0.5 mm wide treatment-affected regions where values were averaged and statistically compared is shaded. Each black dot corresponds to a sampling location on the treated specimen.

In order to perform statistical comparisons of the structural parameters in the treated and untreated corneas, measurements across the microwave treated annulus were averaged across a 0.5 mm distance encompassing the actual 0.25 mm-wide “treated” zone (Fig. 2). Thus, because SAXS patterns were recorded at intervals of 0.25 mm, six data points were averaged per cornea i.e. three on each side of the microwave-treated annulus. The rationale for this approach was that the microwave-induced corneal heating would likely impact upon collagen slightly beyond the immediate treatment zone owing to the effects of thermal conduction within the tissue^28,29^. The 0.5 mm regions are therefore referred to as ‘treatment-affected’ as opposed to ‘microwave-treated”. Corresponding regions of untreated control corneas were similarly analyzed.

## Results

### Axial corneal topography

**Figure 3:**
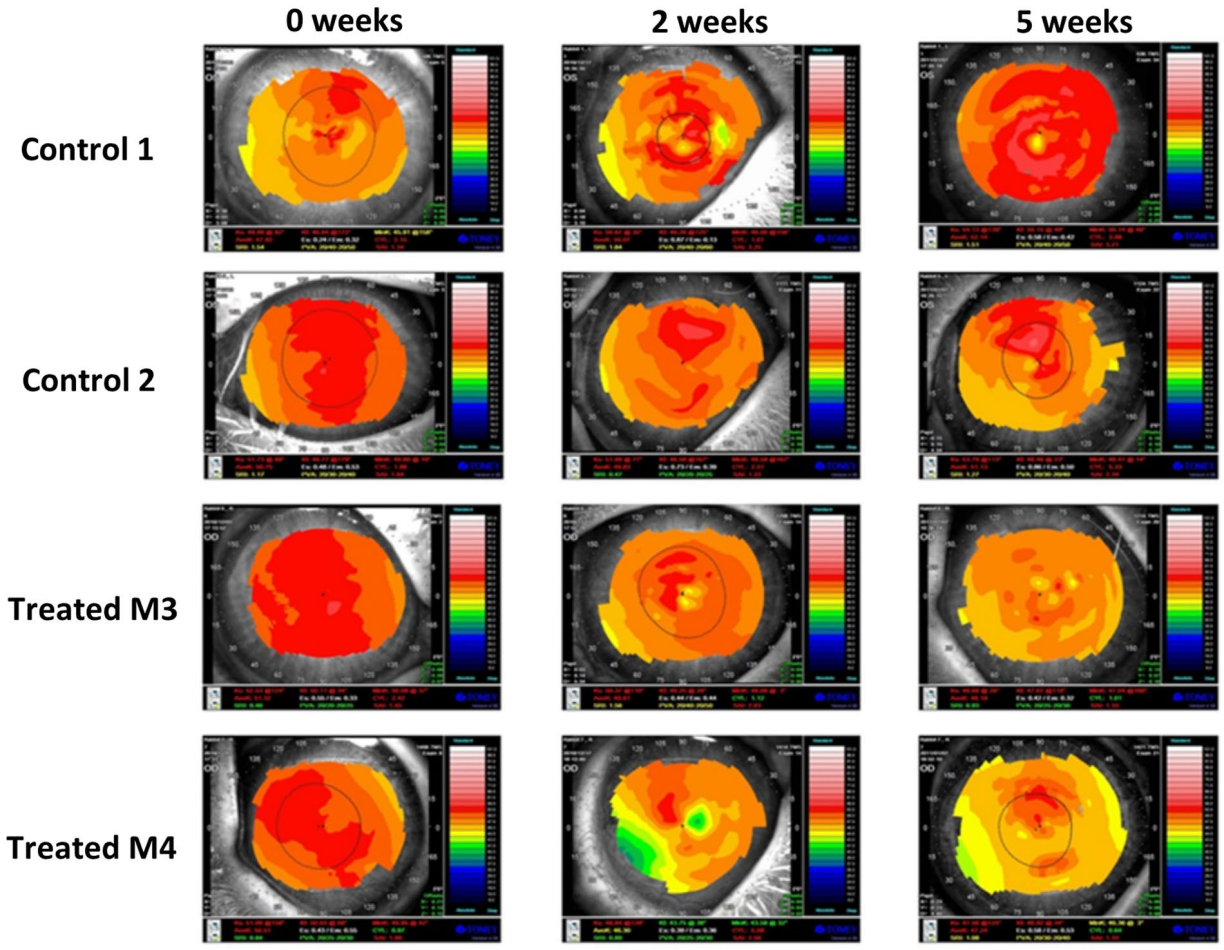
Axial corneal topography maps of rabbit corneas. Maps of pre-microwave treatment (0 weeks) and 2 and 5 weeks post-treatment are displayed (bottom two rows). The maps for control corneas at the same time points are also displayed for comparison (top two rows). The colours visually correspond to “flatness” and “steepness,” with hot colours representing steeper regions of the cornea and cool colours representing flatter regions of the cornea. Variation in surface curvature is evident in both control and treated corneas over the 5 week time frame.

Central corneal topography measurements revealed that microwave treatment resulted in corneal flattening, accompanied by a corresponding change to the cornea’s refractive power. This is reflected in axial topographic maps (Fig. 3) as well as average K values (Table 1). There was some variation in the inter-individual response, however, the overall average K values obtained for microwave treated corneas at two and five weeks post-treatment points to a refractive change of −2.16 D and −2.80 D. At the same time points, control corneas exhibited a refractive shift in the other direction of magnitudes +0.62 D (two weeks) and +2.30 D (five weeks). Unfortunately, corneal topography measurements could only be obtained for two control corneas and, consequently, comparisons for these measurements have been made between four treated and two control corneas.

**Table 1:** Average K values (in dioptres) for control and treated corneas obtained at three time points (one pre-treatment and two post-treatment).

Average K value (D)			
	Pre-treatment	2 weeks post-treatment	5 weeks post-treatment
Control 1*	47.92	50.07	52.14
Control 2*	50.75	49.83	51.13
Average control	49.34	49.95	51.64
SD	2.00	0.17	0.71
Treated M1	50.44	47.62	47.40
Treated M2	49.29	49.19	47.53
Treated M3*	51.32	49.81	48.18
Treated M4*	50.51	46.30	47.24
Average treated	50.39	48.23	47.59
SD	0.84	1.58	0.41

The overall average K and SD values are also shown for both treated and control corneas at each time point. Asterisks (*) indicate the control and treated corneas displayed in Fig. 3.

### SAXS Parameter Profiles

**Figure 4:**
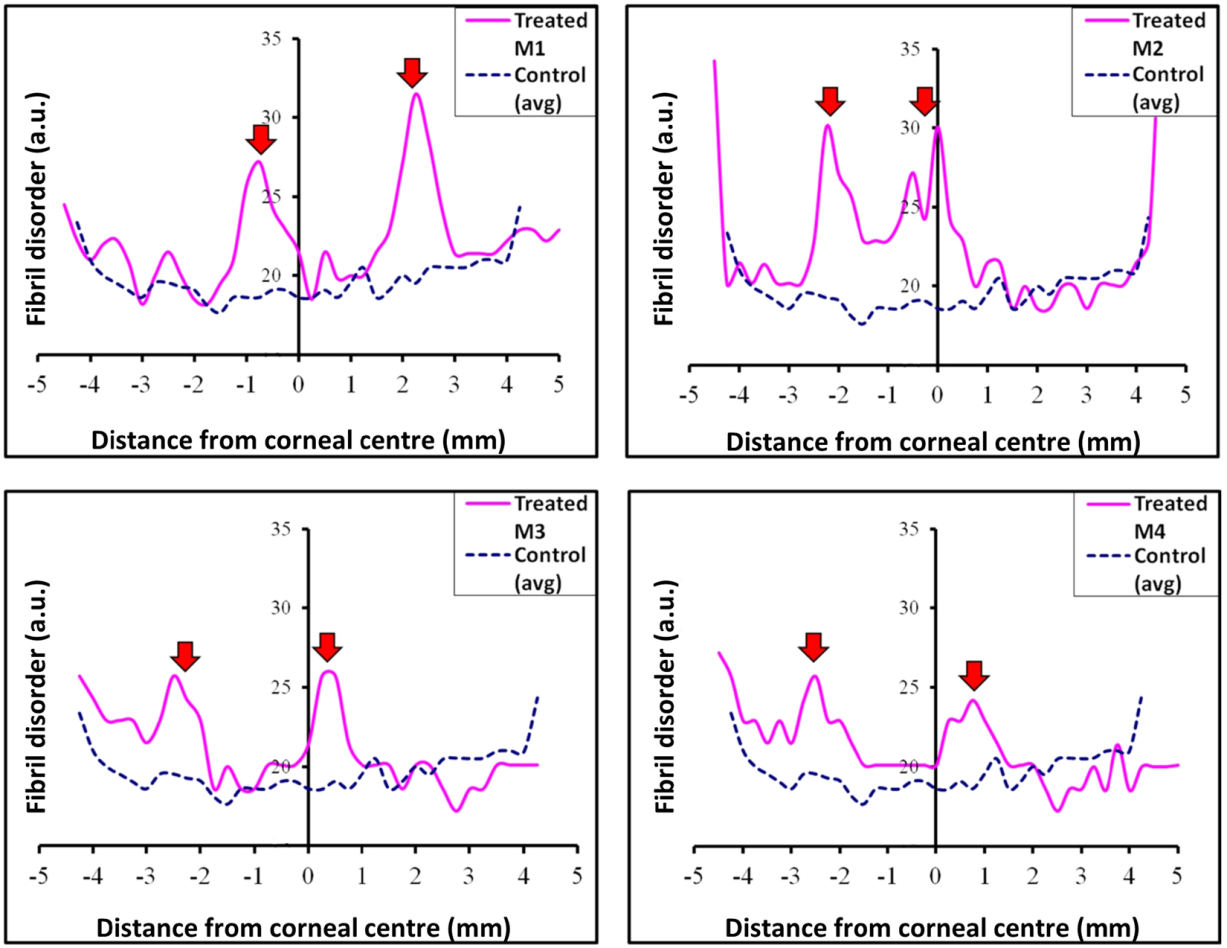
Parameter profiles for fibril disorder across individual treated rabbit corneas. Averaged (n = 3) control values are also shown. Note the marked increase in fibril spatial disorder in the microwaved region for all specimens (red arrows).

SAXS analysis of both treated and control corneas demonstrated the natural increase in fibril disorder that occurs towards the limbus (Fig. 4). Five weeks after microwave keratoplasty a noticeable increase in collagen fibril disorder was observed within the cornea at positions corresponding to the annular microwave treatment zone for all treated specimens (Fig. 4). This loss of spatial order denotes that within these regions of the corneal stroma there was a wider range of collagen fibril spacings than in control tissue. As alluded to earlier, elevation of collagen fibril disorder above normal levels extends beyond the margin of the treatment annulus in some specimens. We propose that this is likely to be due to lateral thermal conduction within the stroma away from the immediate microwave treatment zone^28,29^.

**Figure 5:**
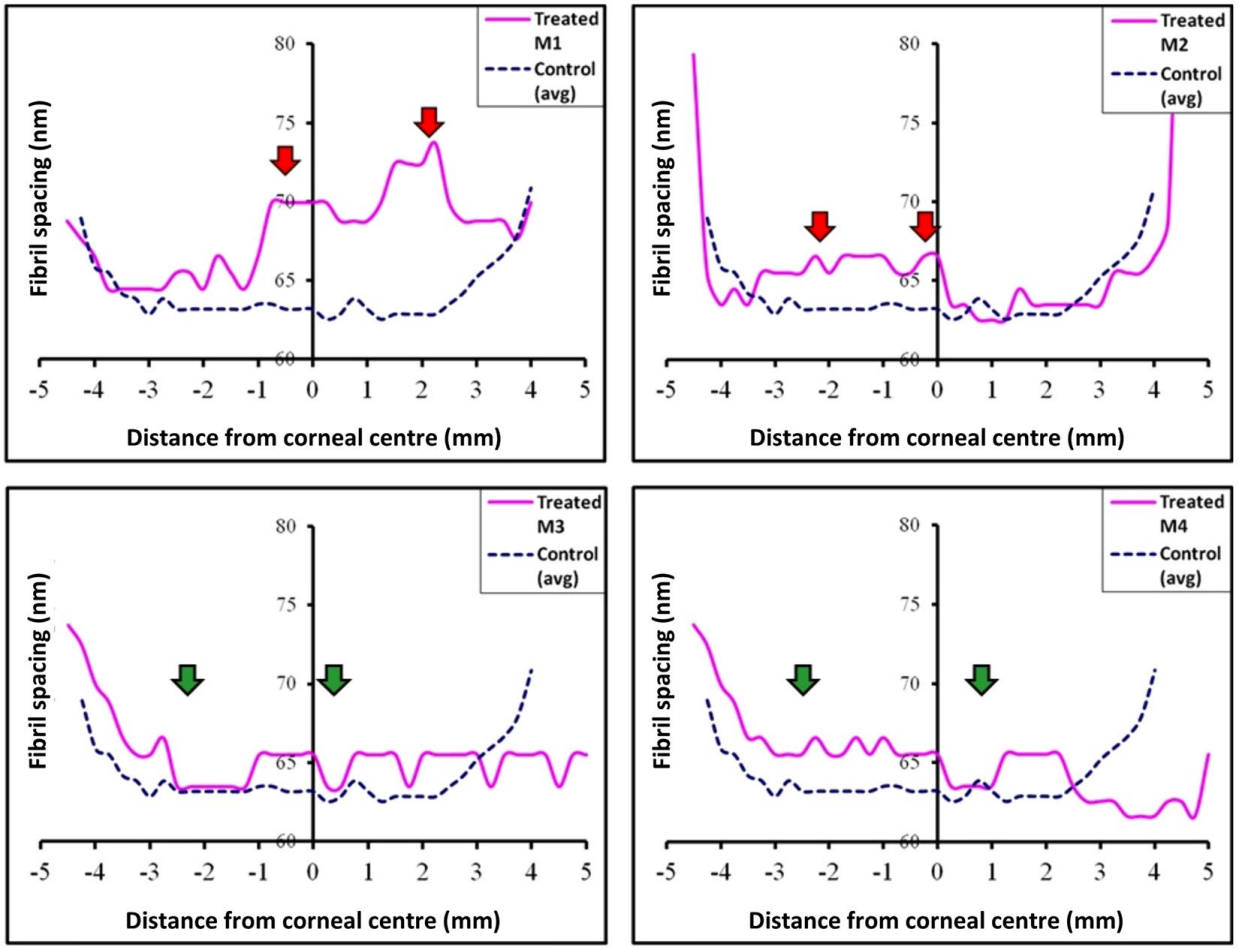
Parameter profiles for fibril spacing across individual treated rabbit corneas. Averaged (n = 3) control values are also shown. Note the increase in fibril spacing for specimens M1and M2 (red arrows). Changes in fibril spacing in the microwave regions of M3 and M4 are less distinct (green arrows).

The SAXS data analysis also indicated an increase in average collagen fibril spacing in two of the four treated specimens (M1 and M2; Fig. 5). As with the peaks observed in the collagen fibril disorder profiles shown in Fig. 4, the heightened fibril spacing above that of untreated controls extended outwardly further than the actual treatment annulus, again most likely as a consequence of thermal conduction^28,29^. Some evidence of an increased average collagen fibril spacing was apparent in treated corneas M3 and M4, but these were less defined than the changes seen in corneas M1 and M2 and were not restricted to the microwaved annulus or the surrounding tissue, thus the changes cannot be directly attributed to the microwave treatment.

**Figure 6:**
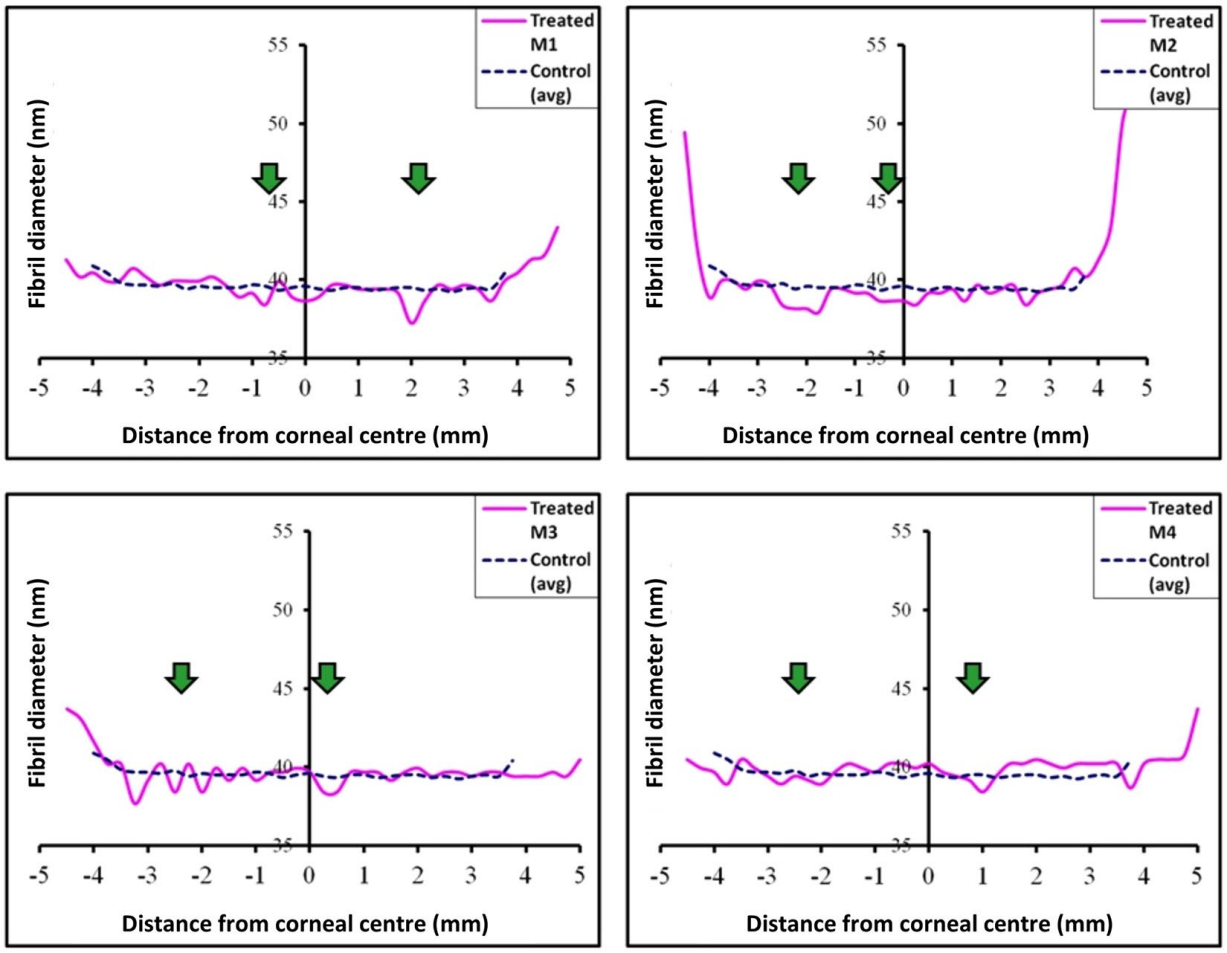
Parameter profiles for fibril diameter across individual treated rabbit corneas. Averaged (n = 3) control values are also shown. No obvious change in fibril diameter is evident within the microwaved region of any of the treated corneas (green arrows).

Interestingly, SAXS analysis of the average collagen fibril diameter disclosed no measurable changes within the microwaved annulus of any of the treated specimens (Fig. 6).

Statistical comparisons of the SAXS-measurable structural parameters within the 0.5 mm wide treatment-affected region described in the Methods section (and shown as the shaded area in Fig. 2) revealed that microwave treatment did not produce any significant measurable changes in either the average collagen fibril diameter (P = 0.069) or the 65 nm axial collagen *D*-period (P = 0.109). Average collagen fibril spacing (P = 0.110) and spatial order (P = 0.003) in the fibrillar array, on the other hand, were markedly altered as a result of microwave treatment, even though the increased fibril spacing did not reach statistical significance owing to variations in the treatment response between animals (SD ± 3.02; Table 2). Thus, it appears as though microwave keratoplasty in rabbit corneas, five weeks after treatment, has more of an effect on structural inter-fibrillar parameters (i.e. collagen fibril spacing and spatial order) than intra-fibrillar measures (i.e. collagen fibril diameter and the axial fibril *D*-period).

**Table 2:** Mean and standard deviation (SD) values for collagen fibril disorder, spacing, diameter and axial *D*-period within the 0.5 mm wide treatment-affected regions (*shaded areas in* Fig. 2).

Fibrillar parameter	Control (n = 3) mean (±SD)	Treated (n = 4) mean (±SD)
Disorder (a.u.)	18.75 (±0.14)*	25.36 (±1.56)*
Spacing (nm)	62.96 (±0.33)	66.39 (±3.02)
Diameter (nm)	39.45 (±0.29)	38.75 (±0.45)
*D*-period (nm)	66.24 (±0.04)	66.16 (±0.07)

Asterisks (*) indicate the significant difference between treated and control specimens at *P* value < 0.01 in two-tailed unpaired t-test.

## Discussion

Unlike other PTK surgeries, microwave keratoplasty aims to induce an annular expansion, rather than contraction, of the treated paracentral zone of the cornea to induce central corneal flattening. *In vitro* experiments in enucleated pig eyes using a prototype microwave thermal keratoplasty applicator to induce a circular treatment zone with inner diameter of 3.2 mm (and an applicator width of 0.7 mm) have provided proof of concept and achieved an average of around 6 D of corneal central flattening (albeit, with a fairly large variation between eyes)^20^. A subsequent *in vitro* study of microwave keratoplasty in six excised human corneoscleral buttons disclosed a mean reduction in curvature in the region of 3 D^3^. Pilot clinical investigation on six human eyes also indicated that a dioptric change of up to 7 D could be accomplished by microwave keratoplasty, but that this regressed and the flattening could not be maintained, even when corneal crosslinking was conducted along with the microwave keratoplasty^30^. A larger, more recent, prospective clinical trial of microwave keratoplasty for myopia in 33 eyes revealed a successful 2–3 D reduction in keratometry 1 month post-operatively accompanied by an improvement in uncorrected visual acuity, but a complete regression of effect thereafter at 3 and 12 months^31^. Similarly, the treatment of 24 eyes with keratoconus jointly by microwave keratoplasty and corneal cross-linking reported that the desired dioptric correction had been achieved 1 month after treatment, only to have regressed by the 12 month timepoint^32^. The fact that the microwave-induced corneal shape change was not maintained, even with the use of proven UV-based cross-linking treatments leads us to suspect that there might be something inherent in the microwave-treated corneal extracellular matrix that renders the cross-linking procedure ineffective, in as much as it fails to stabilise the cornea’s topography. To investigate this, and to understand the effect of the delivery of microwave energy to the cornea more generally, we undertook a structural analysis of the corneal stroma in rabbits, five weeks after microwave keratoplasty.

Previous work has shown that if the temperature of collagen is increased to between 60 and 70 °C then fibrillar shrinkage occurs^20,33^. In the present study, an estimated corneal shrinkage temperature of 65 °C^34^ was achieved in a 360° circular region around the mid-peripheral cornea via an annular application of microwave energy. This resulted in clearly visible circular opacity within the microwave treated area (Fig. 1). Given that the transparency of the corneal stroma is contingent on its constituent collagen fibrils being organized into a lattice-like arrangement with a fairly high level of short-range spatial order^35,36^, we contend that the structural alterations seen in the microwave treated corneal extracellular matrix – the heightened fibrillar disorder, in particular – contribute to the corneal opacification seen in this region of the cornea. Of course, keratocytes in the corneal stroma also allow tissue transparency^37^, and a cellular contribution to the microwave-induced corneal haziness might also occur as a result of heat-induced cell damage, but our x-ray data provide information on extracellular matrix structure only and thus shed no light on this possibility.

The absence of a significant measured change in (i) collagen fibril diameter and (ii) axial *D*-period in the microwave-treated tissue is somewhat confounding. When corneal collagen is subjected to moderate heating, a small number of consecutive hydrogen bonds in the tertiary collagen structure are reversibly disrupted^38^. This occurs without alterations to the primary protein structure, and permits the collagen triple helix to partially unwind and form new cross-links between amino acids^39^. The net effect of this is the linear shrinkage of collagen in a direction parallel to the orientation of the fibrils, with a corresponding expansion in a direction perpendicular to fibril axis^40^. The elevated temperature of the treated tissue will also engender changes to its hydration and cause water redistribution within heated areas and neighbouring areas. With the combination of these effects it would be reasonable to expect to see changes in all four of the structural parameters measured here (i.e. collagen fibril spacing, fibril disorder, diameter and axial *D*-period) following microwave-induced corneal heating. But, this is not the case and collagen fibril diameter and *D*-period remain unchanged.

To try and rationalise this we note that the heating of type I collagen can have reversible and irreversible effects. Severe heating brings about the irreversible transformation of the native triple helical structure into a more randomly arranged coil structure^41,42^, thus impacting on the primary structure and rendering the collagen denatured. It is understood that this transformation occurs primarily via the breaking of the longer sequences of hydrogen bonds that stabilize the triple helix. Ultrastructural studies of thermal shrinkage of collagen in other tissues – following laser treatment of skin, for example^43^ – have shown heat-treated regions to be a mixture of denatured collagen, in which axial *D*-periodic banding is not discernible at all, and of normal fibrillar regions with visible collagen banding. The thermal stability of type I collagen is determined by the concentration of hydroxyproline in an α-helix^44^ and the percentage of reducible cross-links^45^. Perhaps in cornea, fibrils might have a high thermal stability because of the molecular features discussed above. Alternatively, fibrils might represent a mixed population, possessing a range of thermal stabilities, owing perhaps to the relative maturity of the fibril and/or its location in the stroma and past exposure to UV light. When interpreting the current results, it is important to consider that SAXS analysis provides a representative structural average of the whole corneal thickness through which the x-ray beam passes^25,26^. Thus, if a heterogeneous population of variously heat-resistant fibrils existed any change in collagen fibril diameter or axial *D*-period, especially if it occurred in a minority of fibrils, would remain undetected. Microwave treatment has been shown to affect only the anterior half of the corneal stroma of pigs^20^ and humans^3^, however, the affected zone would be expected to be proportionately deeper in the thinner rabbit cornea. Even supposing a larger proportion of tissue is affected in rabbit corneas, we need to remember that when x-ray scattering patterns are analysed it is the modal value of each structural parameter that is the main outcome measure. Thus, it may not be possible to detect a change in diameter or axial D-period, as these structurally altered fibrils may not contribute to the SAXS signal above non-specific background scatter.

The above comments about the unchanged average collagen fibril diameter and axial *D*-period notwithstanding, it is evident that intra-fibrillar changes are seen in the corneal extracellular matrix following microwave treatment, namely a variable increase in average collagen fibril-to-fibril separation and a consistent loss of short-range spatial order in the collagen fibril array. Proteoglycan macromolecules in the corneal extracellular matrix are believed to influence collagen fibrillar organisation via interactions with the fibrils and each other^46–50^. Indeed, SAXS studies have reported a marginal increase in average collagen fibril spacing and a significant loss of fibrillar arrangement in the murine corneal stroma when the keratan sulfate proteoglycans, lumican and keratocan, are genetically altered^27,51^. This type of structural change mirrors that seen here in rabbit cornea following microwave treatment, and it is conceivable that a heat-induced modification of the proteoglycan core protein and/or glycosaminoglycan side chain renders the corneal proteoglycans incapable of interacting with collagen fibrils and, thereby, controlling the structural fibrillar arrangement.

Clinical investigations have shown that the topographic and, therefore, refractive effect of microwave keratoplasty totally recedes, even when corneal crosslinking is carried out soon after the microwave procedure in an attempt to stabilise the cornea and preserve the shape change^30,32^. The mechanism of action by which corneal crosslinking works is still not fully understood, nor is the nature and/or location within the tissue of the cross-links which presumably act to stabilise the cornea^52^. Evidence exists, however, which is consistent with the formation of advanced glycation end products (AGEs) in the corneal matrix^53^. It was proposed that they most likely occur predominantly at the collagen fibril surface and in the protein network surrounding the collagen^54^. Either way, the intra-fibrillar environment is implicated, and this is where we see microwave-induced extracellular matrix changes, documented here. Perhaps, it is the case, therefore, that microwave-induced changes to collagen and/or proteoglycans alters the molecular properties of these tissue components to render current cross-linking modalities ineffective or short-lived.

## Data Availability

The analyzed datasets from this study are available from the corresponding author upon request.
